# Application of disability-adjusted life years to predict the burden of injuries and fatalities due to public exposure to engineering technologies

**DOI:** 10.1186/1478-7954-12-9

**Published:** 2014-03-28

**Authors:** Arun Veeramany, Srikanth Mangalam

**Affiliations:** 1 Public Safety Risk Management, Technical Standards and Safety Authority, Ontario M8X 2X4, Canada

## Abstract

**Background:**

As a public safety regulator, the Technical Standards and Safety Authority (TSSA) of Ontario, Canada predicts and measures the burden of injuries and fatalities as its primary means of characterizing the state of public safety and for decision-making purposes through the use of a simulation model. The paper proposes a simulation-based predictive model and the use of disability-adjusted life years (DALYs) as a population health metric for the purposes of reporting, benchmarking, public safety decision-making, and organizational goal setting. The proposed approach could be viewed as advancement in the application of traditional population health metrics, used primarily for public health policy decisions, for the measurement and prediction of safety risks across a wide variety of engineering technologies to which the general public is exposed.

**Results:**

The proposed model is generic and applicable to a wide range of devices and technologies that are typically used by the general public. As an example, a measure of predicted risk that could result from the use of and exposure to elevating devices in the province of Ontario is presented in terms of the DALY metric. The predictions are further categorized in terms of the causal attribution of the risks for the purposes of identifying and focusing decision-making efforts. The results are also presented by taking into consideration factors such as near-misses or precursor events as termed in certain industries.

**Conclusions:**

The ability to predict potential health impacts has three significant advantages for a public safety regulator – external reporting, decision-making to ensure public safety, and organizational benchmarking. The application of the well-known Monte Carlo simulation has been proposed to predict the health impacts expressed in terms of DALYs. The practicality of the proposed ideas has been demonstrated through the application of the prediction model to characterizing and managing risks associated with elevating devices in the province of Ontario, Canada.

## Background

**Figure 1:**
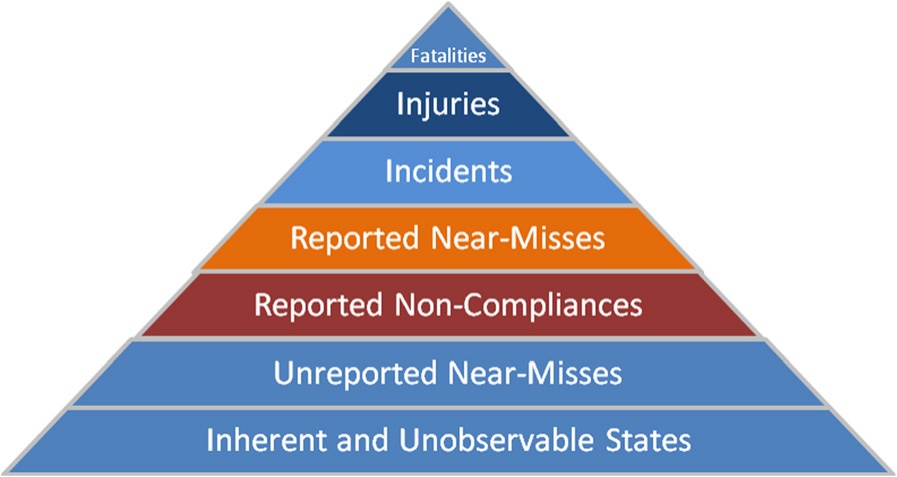
Opportunities for introducing predictive measures.

Information and data on incidents and near-misses are analogous to the visible tip of the iceberg that enables the regulator to be reactive to unfortunate occurrences. There may also be valuable data such as noncompliances obtained typically through regulatory inspection programs that identify underlying or nascent failures in the regulated systems that support proactive decision-making. Additionally, there could be unforeseen scenarios that are best anticipated through a thorough predictive risk assessment. Noncompliances, near-misses, and incidents form valuable predictive inputs to prevent fatalities and injuries. These represent a triangle: the base provides opportunities for improving public safety, and the severity increases as we reach the top in Figure 1. The inherent and unobservable states at the base of the triangle represent faults in the regulated engineering system that are not observed until identified during an inspection or an occurrence is reported.

A judicious choice of a population health metric for the purposes of quantifying risk that is predictive in nature and a risk-acceptability criterion as a benchmarking standard for organizational goal setting are essential. Hence, the objective of this paper is to propose a technique for measuring and quantifying risk in the context of regulated devices and explore the application of disability-adjusted life years (DALYs) as an appropriate metric to predict future health burden.

The TSSA is a not-for-profit self-funded organization that administers and enforces public safety laws in various sectors under Ontario’s Technical Standards and Safety Act. The regulated sectors include elevating and amusement devices, fuels, boilers and pressure vessels, operating engineers, ski lifts, and upholstered and stuffed articles. There are a large number of devices, technologies, and products (herewith known as technical systems and products) with which the public interacts on a daily basis across the province of Ontario. The state of compliance of these technical systems and products to the specific codes and standards is ensured through a collective application of regulatory instruments that span the life cycle of these systems. Active information on the state of compliance is monitored and collected primarily through periodic inspections of the technical systems and sometimes, through random ad hoc inspections. If noncompliances are identified, they are enforced and logged in to an information system. Despite the efforts, there are sometimes incidents and near misses, some of which lead to health impacts in the general public. For the purposes of distinction, an incident may result in a consequence such as an injury or property damage, while a near miss results in an elevated exposure that could potentially lead to such consequences. Incidents and near misses are collectively known as occurrences at the TSSA.

The DALY has been explored by various public safety authorities in Canada and around the world including the Australian Institute of Health and Welfare [1], National Center for Infectious Diseases, USA [2], National Institute for Public Health and the Environment, Netherlands [3], Public Health Agency of Canada [4], Statistics Canada [5], and Health Canada [6]. The TSSA in particular has been using DALYs as a reporting metric since 2010 [7]. A DALY is the loss of a year in an individual’s life who would have otherwise led a healthy life [8]. The intent is to utilize DALYs in a decision-making setting as a single dimensional metric [9] resulting from aggregating morbidity and mortality outcomes. TSSA has already used this approach to risk measurement in prioritizing and managing several known safety issues associated with elevating devices [10].

The concept of using the DALY metric in the future domain has been explored by studies related to the Global Burden of Disease (GBD) [11,12]. Disease-specific projection studies elsewhere have taken inspiration from the GBD literature [13]. These studies have extensively applied multiple linear regression on large amounts of global individual mortality data available to the World Health Organization (WHO). The objective of these studies is to estimate the mortality rate with dependency on various covariates such as smoking, level of education, age. etc. The future burden of disease is then reported in terms of DALYs. The TSSA has unique challenges in the sense that it does not collect in-depth device-specific intrinsic factors, which could potentially be causes of unfavorable human health impacts in the event of occurrences. Some of the factors are age-related susceptibility to degradation mechanisms that accumulate over time, making a device high risk in nature. It is quite common in the engineering failure analysis literature to use this information to predict the state of a device in its future course of usage. As a result of the missing covariate information, the well-explored concept of regression analysis is not applicable for prediction purposes at the TSSA.

However, there is a wealth of credible historic occurrence data along with actual health impacts collected over a period of several years. Hence, the present paper effectively utilizes the available occurrence data in order to predict potential health impacts by implementing a second-order Monte Carlo simulation. The predicted health impacts in terms of DALYs are reported as a range of percentiles so that readers can appreciate the extremities along with the average estimates.

## Methods

### Model overview

**Figure 2:**
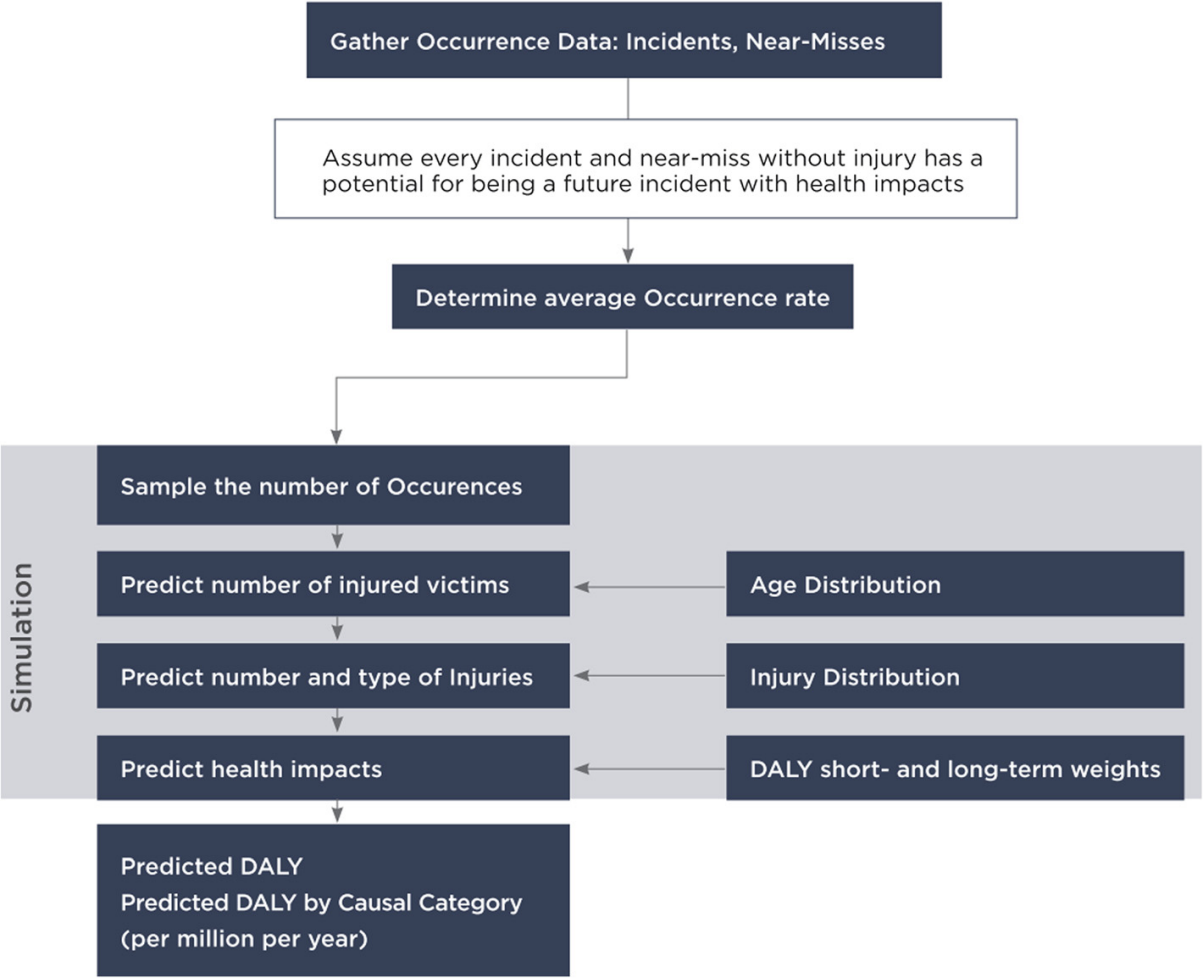
First-Order Monte Carlo simulation (fixed λ) for DALY prediction.

A flowchart of the first-order Monte Carlo simulation for predicting health impacts in terms of DALYs is presented in Figure 2. This level of simulation captures variability in number of victims, injuries, injury types, and age of the victims. However, at this stage uncertainty in the occurrence rate λ is ignored. The end result of the first-order simulation is a DALY distribution classified by causal categories.

Keeping in view the importance of separating variability and uncertainty through a second-order simulation [14], uncertainty in λ could be exclusively dealt with in a second layer of simulation through the reporting bias. This separation increases the model complexity and becomes computationally prohibitive [15].

### Establishing an occurrence rate

The TSSA database has an account of each reported occurrence along with factors such as the date of occurrence, whether the occurrence is an incident or a near miss, the number of injured victims, and number and type of injuries sustained by each victim. Actual or observed DALYs do not account for near misses and incidents without injury. Hence, an occurrence rate based solely on past occurrences with health impacts does not truly reflect what would potentially happen if some of the near misses manifest themselves as occurrences with health impacts in the future. But then the uncertainty lies in how many of the nonhealth impact occurrences turn in to incidents with injuries. We assume two approaches to account for the benefit uncertainty: (1) assume that every such occurrence carries an injury potential in the future (*r* = 1) or (2) assume a near miss to incident ratio based on the safety pyramid rule [16] from the chemical processing industry (*r* > 1). The second assumption has the motivation in the belief that some of the near misses may never have negative health impacts purely by design. For example, activation of relief valves or rupture discs on pressure vessels is designed to manage overpressure scenarios. These activations may be reported as near-miss pressure boundary failures.

Then, the occurrence rate λ is established in terms of the number of incidents *n*_*i*_, number of near misses *n*_*m*_ and the observed duration *T* as:

(1) $$\lambda =\frac{n_{i}+n_{m}/r}{T}yr^{-1}$$

This expression determines the number of occurrences for an iteration of the simulation as a sample from Poisson distribution with λ as the mean occurrence rate. It is assumed that the number of occurrences has been constant over the years. However, it is of future interest to move toward a time-dependent occurrence rate λ(t) so that the number of occurrences in a future year is predictive rather than assuming a past average. A nonhomogeneous Poisson process with an underlying power law model is an alternative.

### Number of injured victims

The number of injured victims in the event of an occurrence is a random variable. This number is dependent on the technical system or product under consideration. For example, in the context of elevating devices, the number could be anywhere between 0 and the maximum observed in the history or the mean number *M* of passengers a typical device could affect. It is assumed that the number of injured victims follows a discrete uniform distribution with parameters *a* = 0 and *b* = *M*. Historically, there have not been more than three injured victims in a single occurrence associated with elevating devices.

### Injury type distribution

**Figure 3:**
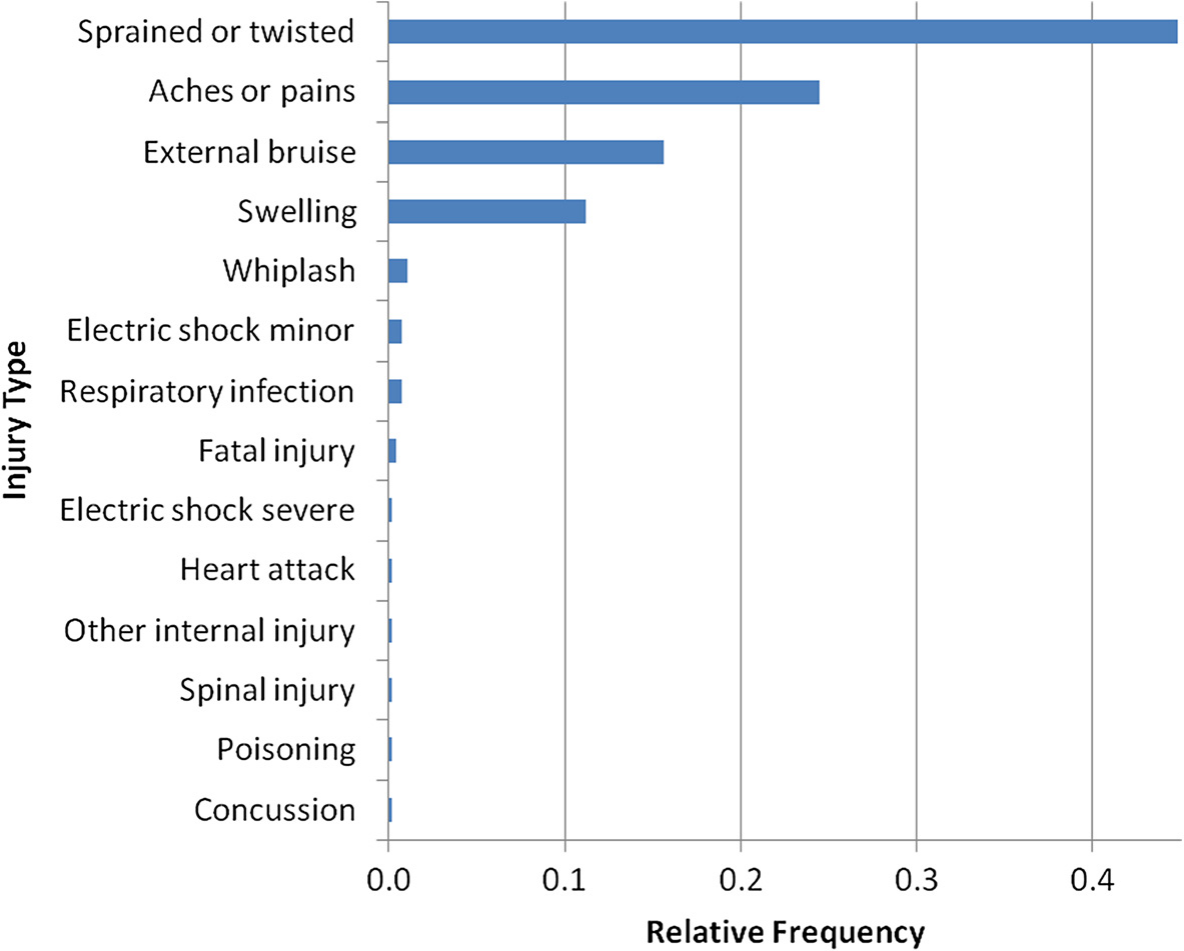
Injury type relative frequency distribution: elevating devices, 2009–2011.

An inspector is usually dispatched in the event of an occurrence to gather circumstantial evidence. The victims, if any, are interviewed for their age and types of injuries sustained and regarding the situation in general. The collected injury information is fed in to TSSA’s Incident Management Information System (IMIS) tool. The historically observed injury type distribution is used for simulation purposes. For example, Figure 3 shows the injury type distribution observed for elevating devices between 2009 and 2011. Though there have been a few fatalities and permanent injuries affecting life expectancy of victims in the usage of elevating devices, the majority of the injuries have been high-incidence, low-disability conditions. A fatal injury equates to death and hence all other injury types are discarded in the event of a death. There could be a few injury types that may not have been observed in the past. In such cases, all the unobserved types are pooled so that the combined frequency sums to one. This ensures that the unobserved types are picked up during simulation but with a lesser chance. The TSSA inspectors choose each of the applicable sustained injury types exactly once during data collection on the field. However, the simulation process samples the types with replacement in order to reduce computational burden.

### Determining the DALY

The expression for calculation of DALYs adopted from the AIHW is given as [1]:

(2) $$\begin{array}{ll} \mathrm{DALY}= & \;\mathrm{Short}‒\mathrm{term}\;{\mathrm{Weight}}^{*}\mathrm{Short}‒\mathrm{term}\;\mathrm{Duration}\\ & \;+\mathrm{Long}‒\mathrm{term}\;{\mathrm{Weight}}^{*}\left(\mathrm{Fraction}\;\mathrm{Long}‒\mathrm{term}\right)\\ & \left({\;}^{*}\mathrm{Long}‒\mathrm{term}\;\mathrm{Duration}\right) \end{array}$$

An injury sustained can have either or both of short-term and long-term health impacts. The weights chosen by AIHW [17] were in turn adapted from the GBD studies at WHO [18]. The long-term duration is the average life expectancy of the victim at the time of the occurrence. For the purposes of simulation, the life expectancy is averaged across gender. Since a long-term disability may not last for the rest of the life, the duration is weighed by the fraction long-term. The assumed weights and durations are listed in Table 1.

**Table 1:** Disability weights related to TSSA specific injury types

**Burden of disease injury type**	**TSSA injury type**	**Short-term weight**	**Short-term duration**	**Fraction long-term**	**Long-term weight**
Intracranial injuries [17]	Concussion	0.359	0.0671	0.05	0.35
Poisoning [17]	Poisoning	0.611	0.0082	0	0
Injured spinal cord [19]	Spinal injury	0.725	0	1	0.725
Sprains [17]	Sprained or twisted	0.064	0.0384	0	0
Internal injuries [17]	Other internal injury	0.208	0.0425	0	0
Upper respiratory infections – pharyngitis [19]	Respiratory infection	0.07	0.02	0	0
Rheumatic heart disease and heart failure [20]	Heart attack	0.323	0.1	0.2	0.353
	Aches or pains	0.02	0.02	0	0
	Electric shock minor	0.04	0.02	0	0
	Electric shock severe	0.2	0.1	0.1	0.15
	External bruise	0.04	0.02	0	0
	Swelling	0.04	0.02	0	0
	Whiplash	0.04	0.02	0.05	0.04
	Fatal injury	0	0	1	1
	No Injury	0	0	0	0

A DALY estimate from Equation (2) for fatality of an individual is determined by setting the short-term parameters (disability weight and duration) to zero, the long-term parameters (disability weight and fraction) to one, and long-term duration as the remaining life expectancy of the deceased. The result is a DALY value which is equal to the victim’s expected life expectancy at the age of death. In a general setting, if the remaining life is set to median life expectancy in Ontario (44.4 years; subject to change), the resulting DALY associated with mortality could be used as a benchmark and guiding principle for decision-makers.

### Adaptation of AIHW disability weights to specific TSSA injuries

The injuries observed from occurrences resulting from TSSA-regulated technologies (Table 1) do not correspond exactly with the injury types in the AIHW tabulation [17]. Where the TSSA-defined category did not evidently correspond to one from the Victorian study, the most appropriate proxy was considered, e.g., intracranial injuries for concussion. If a certain TSSA injury type was associated with subcategories in other studies, the most conservative one was chosen. As an example, for respiratory infections, the disability weight associated with pharyngitis was chosen from the GBD 2004 update [19]. Some injury types such as heart attacks have been reported to the field inspectors in the past. These are possible health outcomes from several scenarios such as entrapments, electrical shocks, falls, etc. during elevator rides and hence have been included in the list of injury types. Rheumatic heart disease was chosen for short-term disability and heart failure was chosen as the long-term one [20]. In case of fatality, disability weight and fraction of long-term duration are both assigned a one so that the DALYs are set to the life expectancy of the victim. All parameters are set to zero for “no injury” in order to keep track of the number of occurrences where there was no injury. TSSA regulates various sectors, the injury types across which range from minor injuries to permanent ones, including mortality. The current choice of injury types is subject to revision based on shortcomings identified [21] in relation to differentiating high-incidence low-severity injuries from low-incidence high-severity injuries and also based on the improved methodology to measure disability weights [22]. As a proof of concept, high-incidence injury types (e.g., aches and pains) that do not correspond to the GBD study have been assigned a very low disability weight (< 0.02) and an approximate duration of 1 week (< 0.02). These assumptions are needed until a more robust theory is articulated on determining DALYs for injuries rather than for diseases. The authors are of the opinion that public safety decision-making in sectors dominated by injuries is better informed with certain assumptions for disability weights, although some of them cannot be directly mapped to the burden of disease studies. This scheme is more informative than making decisions solely based on actual number of observed infrequent fatalities.

### Occurrence cause classification

It is believed that incident and near miss occurrences are the ultimate realization of safety risks and can result from any one of three causal safety impact measurement (SIM) categories:

• SIM 1 – causal factors related to potential inadequacies in available regulatory controls

• SIM2 – causal factors related to noncompliance with current regulatory controls

• SIM 3 – external causal factors not within the purview of current regulatory controls (e.g., behavioral factors, weather conditions, etc.)

Occurrences that do not have an established root cause after inspection are contained in a fourth category, root cause not established (RCNE).

**Figure 4:**
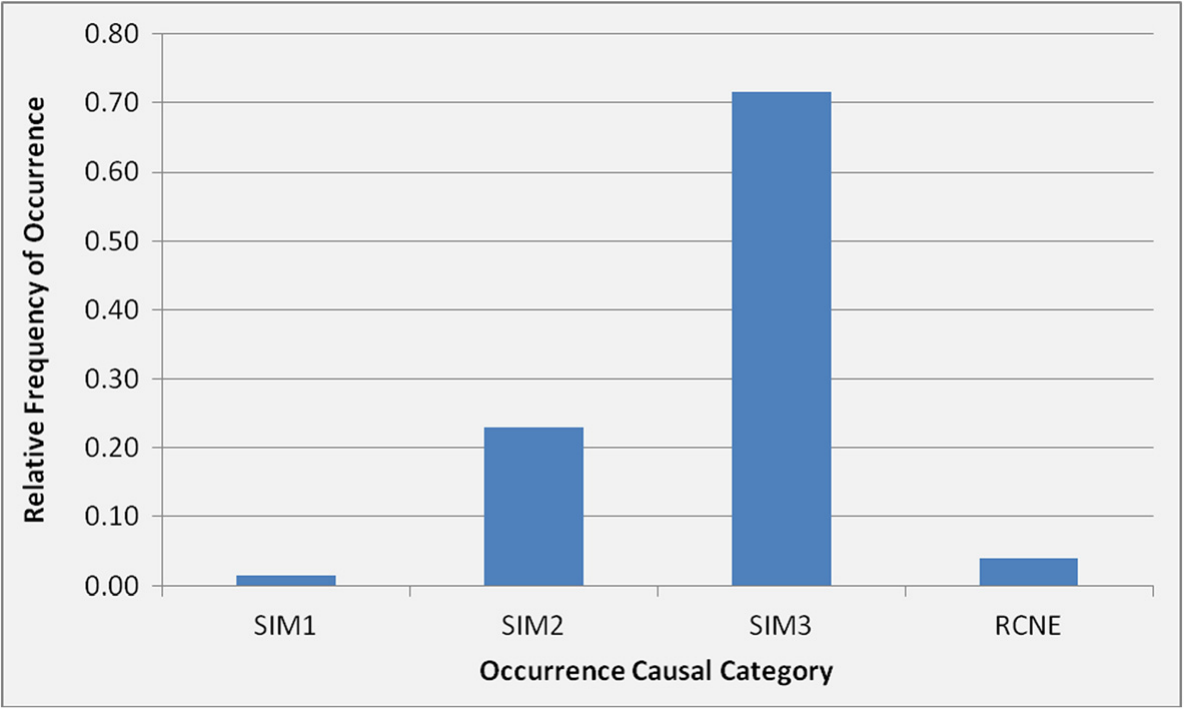
Relative frequency distribution for causal categories.

For each simulated occurrence, one of the causal categories is sampled based on a relative frequency distribution of past observations. For example, Figure 4 shows the distribution for the case of elevating devices observed over the past 4.6 years.

There are sectors that TSSA regulates in which high-incidence low-disability conditions are quite common. In such circumstances, TSSA continues to focus on frequency of system-induced failures and causal reasoning alongside the risk knowledge driven by the DALY metric in order to undertake strategic public safety decisions.

## Results

### Application to elevating devices

**Figure 5:**
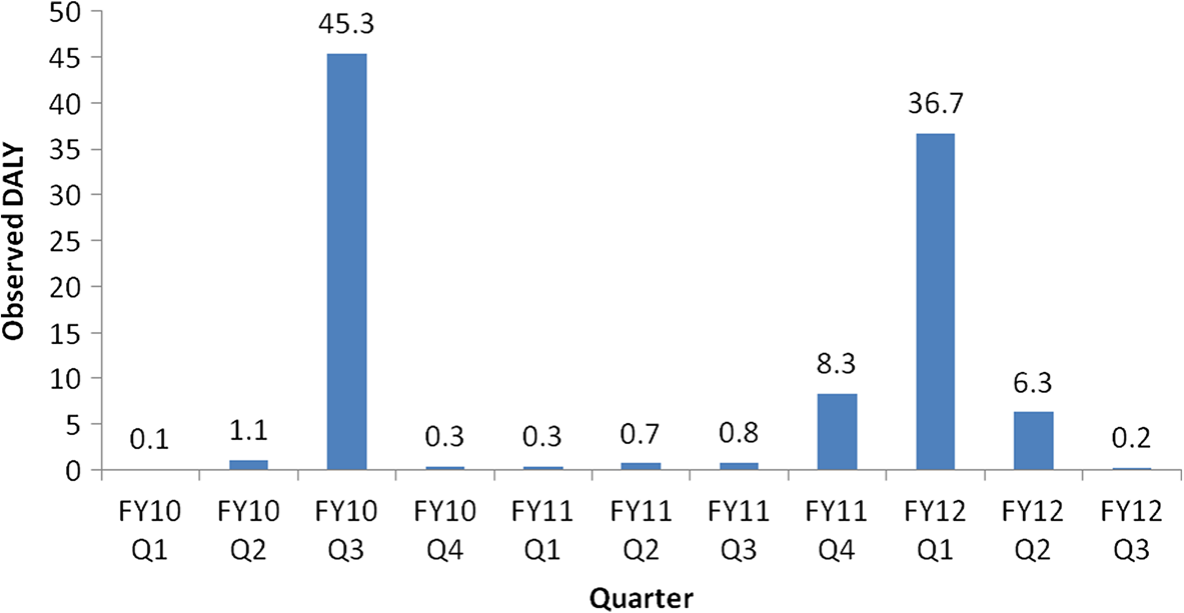
Historically observed DALY in each quarter: elevating devices.

**Figure 6:**
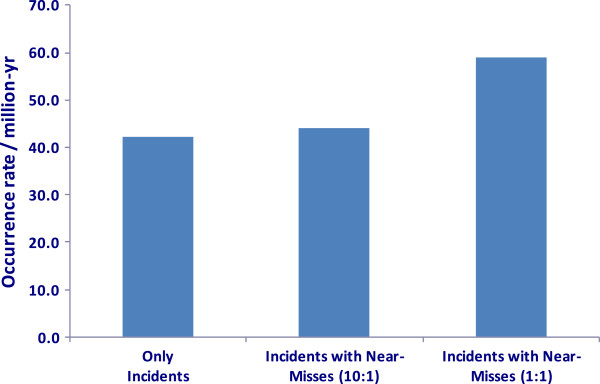
Occurrence rate with and without near misses.

**Figure 7:**
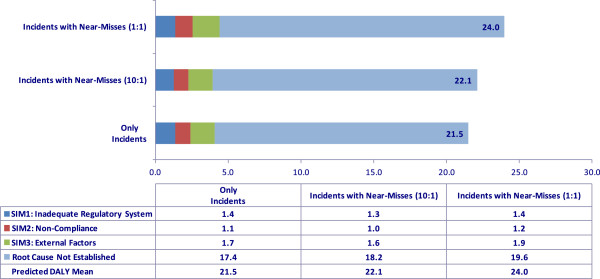
Predicted DALYs without reporting bias.

**Figure 8:**
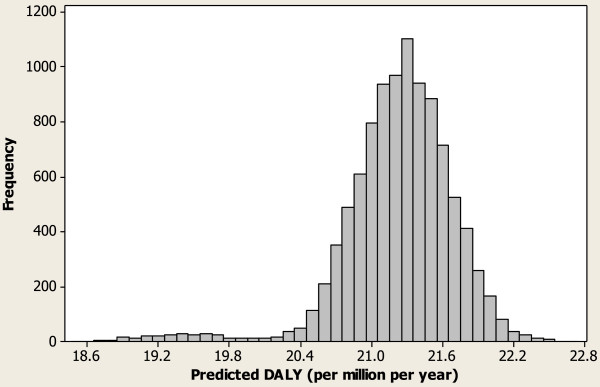
Predicted DALY distribution without near misses and reporting bias.

The proposed model was implemented to understand the risks associated with the use of and exposure to elevating devices in the province of Ontario on a pilot basis. There are over 48,000 elevating devices including escalators in the province of Ontario with a susceptible-to-risk population base of about 13.4 million people. The assumption is that every Ontarian is equally exposed to the risk of an elevating device occurrence at some age in their lifetime. The reported occurrences were *n*_*m*_ = 1028 near misses and *n*_*i*_ = 2638 incidents (2,500 with injuries and 138 without injuries). Experts at TSSA believe that nearly 10 occurrences go unreported for each reported occurrence. It was assumed that there could be anywhere between zero and six riders in a typical elevator and that each injured victim could sustain up to four injuries. As a frame of reference, Figure 5 shows the actual observed DALYs in the past *T =* 4.6 years. The mean occurrence rate represented in terms of a million people per year is shown in Figure 6 for the cases where *n*_*m*_ *=* 0, *r* = 10, and *r* = 1. *r* = 1 has been used at TSSA for reporting purposes which conservatively assumes that every near miss has a potential to result in a future incident with injuries. *r =* 10 does not have a strong basis to defend, however serves as a sensitivity analysis in case only one out of 10 near misses results in incidents. The simulation was run to yield mean DALYs in the absence of reporting bias. The results are shown in Figure 7 categorized by the SIM classification. While the average of the prediction has been selected for reporting purposes, the simulated DALYs form an empirical distribution. A single case with near misses and reporting bias omitted is shown in Figure 8.

## Discussion

In the context of elevating devices, there have been four fatalities observed in the past, and the predicted expected health impact is about 22 DALYs per million people per year in the absence of reporting bias. DALYs for the cases without near misses and with *r* = 10 in Figure 7 are the same (0.5), signifying the possibility that though there are apparently many near misses happening in the province of Ontario, only a few are being actively reported to TSSA. Assuming a one-to-one ratio between incidents and near misses, the DALY estimate is 26.6. The predicted SIM3 risk (1.9 DALYs) indicates that occurrences related to elevators in Ontario are dominated by user behavior and factors external to the regulatory system. A number of occurrences under RCNE were recategorized at the end of the year as user behavior-related. In order to reduce risk related to aging systems that do not conform to the current elevator codes, the elevator risk reduction group analyzed elevators with single- and two-speed motion control and the associated risks to passengers when the elevators do not stop at the prescribed level. This analysis could lead to a replacement of older control systems in the near future. In order to tackle public safety risk due to noncompliance, TSSA issued a communication to elevating device owners reminding them of their legal responsibilities with regard to elevator maintenance and oversight of their maintenance contractors. The owners were advised of new requirements and the timeline in which they will become effective so they can better plan for improved compliance. Figure 8 shows the predicted health impacts as a distribution to illustrate the range of health impacts predicted in the elevating devices sector.

The proposed model supports risk-informed decision-making and hence has a potential to influence policy implications at the organizational level. In particular, the model has significant importance for technical systems, such as those involving high pressure boilers and vessels, where there is possibility of a high-consequence, low-probability incident with health impacts to the population, even though most of such occurrences are rare events making it difficult for policymakers to take risk-informed safety initiatives. Secondly, the model could be explored for new technologies being brought in to the purview of the regulatory system for which there is no health impact data or where there is an effort to strengthen the data management system of an existing technology. While the proposed model strives to bring predictive power to risk assessments, traditional systems analysis of engineering devices based on failure frequencies continues to additionally inform recommendations for public safety decision-making.

## Conclusions

The present paper explored the application of DALYs, traditionally used as a population health metric, for purposes of measuring and quantifying public safety risk in the context of engineering devices and technologies. In the absence of extensive covariate information, it was shown that using a Monte Carlo simulation, technology-induced health impacts could be predicted for the future. The simulations combine mortality and morbidity to yield future health impacts (or public safety risks) in terms of DALYs expected in a given year. The categorization of DALYs into causal factors allows for policy-setting and resource allocation. The proposed model was demonstrated in the context of characterizing and managing public safety risk for elevating devices in the province of Ontario, Canada.

## Competing interests

We hereby declare that we do not have competing interest.

## Authors’ contributions

All the authors took equal responsibility in thoroughly proofreading the manuscript. SM proposed the idea of applying DALYs as a health metric for prediction purposes. SM and AV devised the prediction model. AV implemented the model. Both authors read and approved the final manuscript.
